# Ultrasonically synthesized MOFs for modification of polymeric membranes: A critical review

**DOI:** 10.1016/j.ultsonch.2022.106202

**Published:** 2022-10-14

**Authors:** Amirhossein Taghipour, Ahmad Rahimpour, Masoud Rastgar, Mohtada Sadrzadeh

**Affiliations:** Department of Mechanical Engineering, 10-367 Donadeo Innovation Center for Engineering, Advanced Water Research Lab (AWRL), University of Alberta, Edmonton AB T6G 1H9, Canada

**Keywords:** Sonication, MOFs, Mixed matrix membranes, Surface modification

## Abstract

Metal-organic framework (MOF) membranes hold the promise for energy-efficient separation processes. These nanocrystalline compounds can effectively separate materials with different sizes and shapes at a molecular level. Furthermore, MOFs are excellent candidates for improving membrane permeability and/or selectivity due to their unique properties, such as high specific area and special wettability. Generally, MOFs can be used as fillers in mixed matrix membranes (MMMs) or incorporated onto the membrane surface to modify the top layer. Characteristics of the MOFs, and correspondingly, the properties of the MOF-based membranes, are majorly affected by their production technique. This critical review discusses the sonication technique for MOF production and the opportunities and challenges of using MOF for making membranes. Effective parameters on the characteristics of the synthesized MOFs, such as sonication time and power, were discussed in detail. Although the ultrasonically synthesized MOFs have shown great potential in the fabrication/modification of membranes for gas and liquid separation/purification, so far, no comprehensive and critical review has been published to clarify such accomplishments and technological gaps for the future research direction. This paper aims to review the most recent research conducted on ultrasonically synthesized MOF for the modification of polymeric membranes. Recommendations are provided with the intent of identifying the potential future works to explore the influential sonication parameters.

## Highlights

• Ultrasonic cavitation phenomena and its benefits for production of MOFs is briefly explained.
• Application of MOFs for modification of polymeric membranes is highlighted.
• The current research gap, challenges and future outlook are presented.

## Graphical abstract

**Figure:**
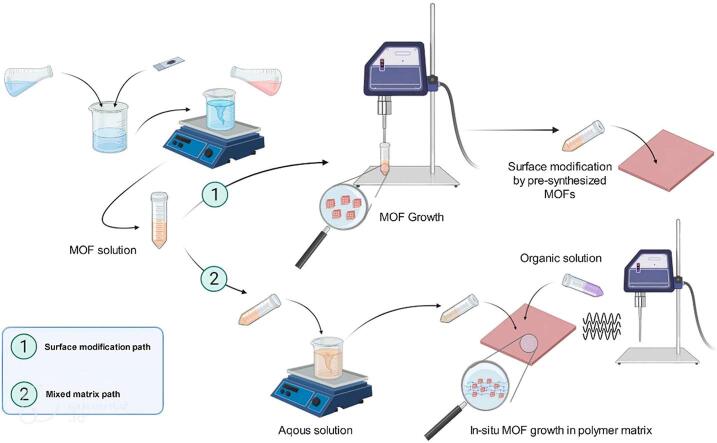

## 1 Introduction

Industrial emissions cause immense environmental problems by contaminating natural resources on earth. Air pollution and water contamination are major dilemmas threatening human existence, not only due to environmental issues but also many negative impacts on social and personal health with increased sicknesses and death [1]. Several contaminants are substantial contributors to human illness. For instance, micro/nano-size particles enter the respiratory system by breathing and cause cardiorespiratory diseases, nervous system malfunction, and cancer [2]. Besides, contaminated water endangers public health, affects food security, degrades ecological processes, and suppresses social prosperity [3]. The presence of emergent contaminants such as medicines, pesticides, and commercial and chemical products, has aggravated existing water discharge problems [4].

Based on the literature, adsorption [5] and gas membranes [6] have been extensively utilized for the separation of air pollutants (greenhouse gases (GHGs) such as CO_2_) from the atmosphere. For water purification, membrane technology is a reasonably stable, adaptable, and reliable option. It offers significant benefits such as easy installation and regeneration, low energy consumption, and the capacity to separate various chemicals [7]. Membranes as selective barriers only allow permeation of certain molecules or ions through while obstructing the easy passages of others. In general, the driving force in the membrane-based separation processes is the chemical potential gradient between two sides of the membrane as a result of hydraulic/osmotic pressures [8], [9], [10], electric field [11], [12], or temperature differences [13]. Fouling and trade-off between permeability/selectivity are long-lasting challenges in membranes technologies [14]. For instance, in water treatment, surface hydrophobicity/hydrophilicity is a crucial factor in membrane fouling [15], [16]. Numerous attempts have been made to resolve this issue by modifying membrane’s active layer [17]. Specifically, incorporating hydrophilic functional groups into polymeric membranes has considerably enhanced their performance [18]. Modification of the active layer via different techniques like layer-by-layer (LBL) assembly, chemical grafting, and coating (e.g., dip coating, spin coating, and electrospinning) have been widely practiced [19], [20]. Membranes have also been modified with various emerging nanomaterials, such as carbon nanotubes [13], [21], graphene oxides [22], [23], [24], [25], and nanodiamonds [26], [27]. While these nanomaterials improved membrane performance by enhancing their selectivity, hydrophilicity, and fouling resistivity, their incompatibility with polymer matrix, and thus releasing during the filtration process, raises concerns regarding the robustness and sustainability of the resulting membranes. This has turned the attention of researchers to either further functionalization of nanomaterials to make them more compatible with polymers or utilizing nanomaterials having both organic and inorganic moieties. Metal-organic frameworks (MOFs) MOFs are a group of crystalline porous materials consisting of metal ions or clusters interconnected by a variety of organic compounds [28], [29], [30], [31], [32], [33]. The presence of organic ligands in MOFs can potentially improve their compatibility with the polyamide matrix when compared to other inorganic nanomaterials [34].

The highly porous structure and tunable chemical functionality of MOFs make them promising materials for water treatment and gas separation. Their super porous network, with stable pores during the solvent elimination process, resulted in unique one, two, or three-dimensional nano-structures. From the first successful report on these materials by Yaghi and his coworkers [35], around 90,000 different MOFs have been designed and synthesized by 2020 [36]. Special features, including regular and tunable pore structures, processability, abundant adsorption sites, and structural diversity, widened the application of MOFs in sensors [37], catalysts [38], gas storage [39], membrane-based separation [40], and antibacterial application [41], [42], [43]. So far, various techniques such as conventional slow endothermic diffusion [44], hydrothermal [45], solvothermal [46], microwave (MW) [47], electrochemical [48], and many others have been introduced for MOF synthesis. The heating-based techniques (generally electrical heating) are usually carried out in a batch system running at mild temperatures below 250 °C and atmospheric pressure. Although thermal methods have been applied in many pieces of research to synthesize MOFs, they suffer from severe challenges like long reaction time (several hours to a few weeks) and high reaction temperature. Recently, some innovative techniques such as ion-thermal technique [49] and room temperature synthesis were reported to produce high-quality MOFs on a big scale [50], [51]. Microwave-assisted MOFs synthesis technology benefits from many advantages, including short reaction time, the possibility of internal heating in batch systems, yields with smaller crystalline size, and phase selectivity [52], [53]. Sonochemical techniques provide many outstanding features, including short reaction time, low operating temperature and pressure, ease of control, and high-quality products [54], [55]. The massive released energy from the explosion of microbubbles led to the formation of hot spots, which facilitates the rapid crystallization of MOFs at ambient temperature and pressure [56]. Ultrasonic-assisted MOFs with remarkable characteristics for membrane technology have been introduced so far [57].

The membranes modified with MOFs illustrate better selectivity and permeability features with minimum structural defects on the matrix than the pristine membranes [58]. Additionally, compared to inorganic fillers such as zeolites, controllability over the pore size and structure is more facile in MOFs [59]. For instance, MOFs with a hydrophobic nature have a strong affinity toward organic substances, making them a great candidate for removing organic contaminants from water/wastewater using the pervaporation technique. The concentration, size, and shape of MOF determine the type of its interaction with the membrane matrix. High MOF loads may cause particle aggregation, leading to water flux reduction and membrane swelling. Besides, some recent research indicated that MOFs with smaller sizes are more compatible with the host polymers [60]. The incorporation of the ultrasmall MOFs particles into the polymer matrix effectively reduced the formation of non-selective microvoids around the particles and enhanced substantially the membrane selectivity [61]. It has been revealed that ultrasonic irradiation can reduce MOF crystal size, preventing permeability reduction by incorporating nanomaterials into the polymer [62]. To the best of our knowledge, there is no review investigating the potential application of ultrasonically synthesized MOFs for membrane fabrication and modification. In the first part of the present review, some recent studies investigating the effect of sonication parameters on the properties of MOFs are summarized. While the main focus of the second part is on the polymeric MOF-membranes in which sonication technique was utilized for production of MOFs.

## 2 Metal-organic frameworks

**Fig. 1:**
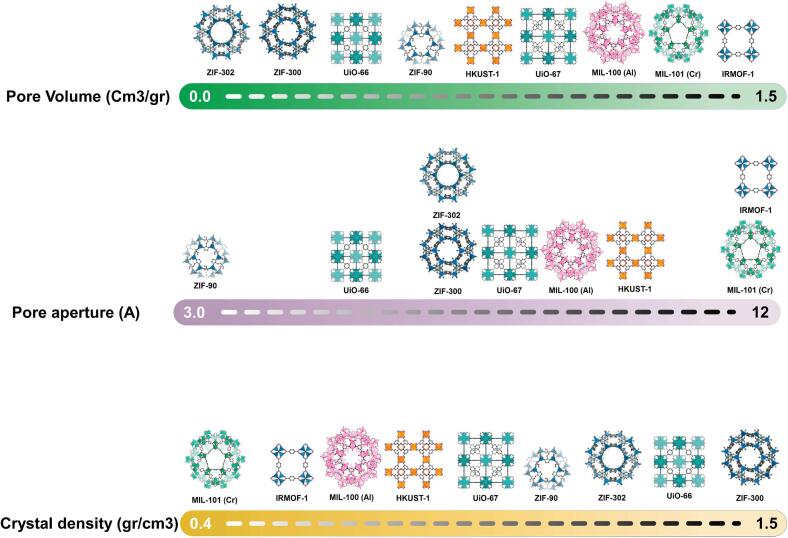
MOF structures and corresponding parameters of some mostly used MOFs in membrane-based gas separation processes (reproduced with permission [57]).

During the past 25 years, the discovery of MOFs as highly porous nano-scale crystalline structures spontaneously attract attention for their surprisingly high surface area (up to 7000 m^2^/g), adjustable pore aperture, and stability (mechanical/thermal). These noncrystalline materials have been utilized for various applications such as gas storage, gas sensors, gas separation, membrane modification, catalysis, drug delivery, etc. [63], [64], [65], [66], [67], [68]. A list of the most frequently utilized MOFs for gas separation applications and the corresponding crystalline structures is presented in Fig. 1. As can be observed, by choosing different metal nodes and organic ligands, the volume and aperture of the pores can be engineered precisely. Generally speaking, MOFs are formed in a coordination reaction between a transition metal ion and an anionic organic ligand (mostly carboxylate functional groups). The resulting nanocrystalline structures are highly porous and pose a surprisingly large surface area favoring their application in advanced material science. Due to their fixed and tunable pore size, MOFs have been utilized in size-sieving-based separation processes in the form of pristine MOF sheets or composite materials [69], [70]. The synthesis of MOF sheets is subjected to some challenges, including surface defects and cracks. Consequently, their application in separation processes has been commonly explored as fillers in membranes (MOF-based mixed matrix membranes). In an ideal scenario, strong interactions between membrane materials and MOFs could dramatically increase membranes' transport characteristics and processability [71]. MOFs can be classified based on their structural features, which is a major factor in their potential application. Rigid MOFs are those with high stability under harsh environmental conditions and are usually utilized for molecular sieving. In contrast, the shape of flexible or dynamic MOFs easily changes by changing some environmental stimuli.

### 2.1 MOFs synthesis techniques

MOFs generally consist of two types of building units, primary building units (PBUs) which are the metal ions and organic compounds, and secondary building units (SBUs) which refer to metal–oxygen-carbon clusters with intrinsic geometric topology. In order to form coordination bonds, the organic ligands always contain functional groups such as carboxylate, nitrile, amine, etc. The first reported techniques for MOF synthesis were carried out under relatively mild conditions [72]. However, the next class of synthesis techniques was based on the temperature-driven solvent-assisted method. The most crucial parameter in thermally-coordinated MOFs is the reactor's temperature, which directly determines their morphology. More recently, some other techniques, including microwave-assisted, electrochemical synthesis, sonochemical synthesis, and layer by layer technique, have been introduced for MOF synthesis [63]. The crystallization route in these techniques is different from the conventional methods, which thoroughly affects the reaction time and the features of the final products. The controllability over particle size distribution, morphology, and crystallization time in microwave-assisted techniques has been reported in the literature [73]. The metal source for coordination with ligands is continuously released from the anode in the electrochemical pathway. The layer-by-layer technique is utilized to produce MOF films by immersing a functionalized pristine sheet in aqueous media containing metal ions and ligands [74]. The sonochemical synthesis of MOFs is conducted by the released energy from the explosion of microbubbles on a microscale. It was shown that the reaction time and crystal size could significantly decrease by using the sonosynthesis technique [75].

Subsonic and ultrasonic waves are longitudinal mechanical acoustic waves that are generated via pressure phase changes in a medium [76], [77], [78]. The frequency of generated sound waves is the pressure change per unit of time where ultrasounds have a frequency higher than 18 kHz [79]. The propagation of ultrasonic waves in an elastic medium, such as a liquid, starts a cycle in which alternative compression and rarefaction occur adiabatically [80]. Compression and rarefaction cycles increase and decrease the molecular distance, respectively, as long as the pressure amplitude reaches the tensile strength, leading to the appearance of tiny cavities [81]. Afterward, the microbubbles grow to reach their critical size and finally collapse. Perusich and Alkire [82] equations are generally used to calculate the released energy in ultrasonic cavitation phenomena:

(1) $$E_{u}=\frac{I}{C}\pi a^{2}\left(2\xi \right)$$

where ξ,a,I and C are acoustic particle displacement amplitude (cm), transducer radius (cm), intensity (W/cm^2^), and speed of sound (cm/s), respectively. By substituting the presented value for ξ and I in eq. (1), an expanded expression of the total energy emitted from the ultrasonic transducer can be presented as follows:

(2) $$E_{u}=\frac{2}{C\omega }{\left[\frac{\eta \pi^{5}a^{4}W_{c}I^{4}}{4\;\rho \;I_{c}}\right]}^{1/3}$$

which ω, η, $W_{c}$, ρ, and $I_{c}$ are angular wave frequency (rad/s), transducer efficiency, calibration power (W), density (g/cm^3^), and calibration intensity (W/cm^2^), respectively. The explosion of microbubbles leads to a quick energy emission on a microscopic scale, which results in sonoluminescence phenomena [83]. This appears as an extreme rise in local temperatures and pressures up to 5000 K and 1000 atm, respectively, followed by very fast cooling rates [84], [85]. The breakdown of molecule bonds is expected in such a harsh condition (e.g., water molecules could disintegrate into hydroxyl radicals and hydrogen [86]). Acoustical variables, including frequency and intensity and pressure, temperature, and pH of the liquid, have been considered the most influential parameters in US-assisted processes. For instance, the average size of ultrasonically-driven cavities at lower frequencies results in more violent cavitational collapses [87]. The intensity of sound waves will increase the acoustic pressure amplitude, causing a more violent cavitational effect [88]. The energy effects of ultrasonic cavitation and, consequently, extreme turbulent movements arising from microjets and microstreaming phenomena may significantly accelerate some physical processes [89], [90]. Enhancing processes such as melting [91], mixing, desorption, extraction, and cleaning [90] are examples of ultrasonic cavitation effects in physiochemical properties. The unique characteristics of the ultrasonic cavitation phenomenon are greatly favorable for rapid synthesis processes, especially those which should be carried out at low temperatures. For instance, sonication was utilized for rapid crosslinking of fluorescent amphiphilic copolymers for the first time by Guo et al. [92].

**Fig. 2:**
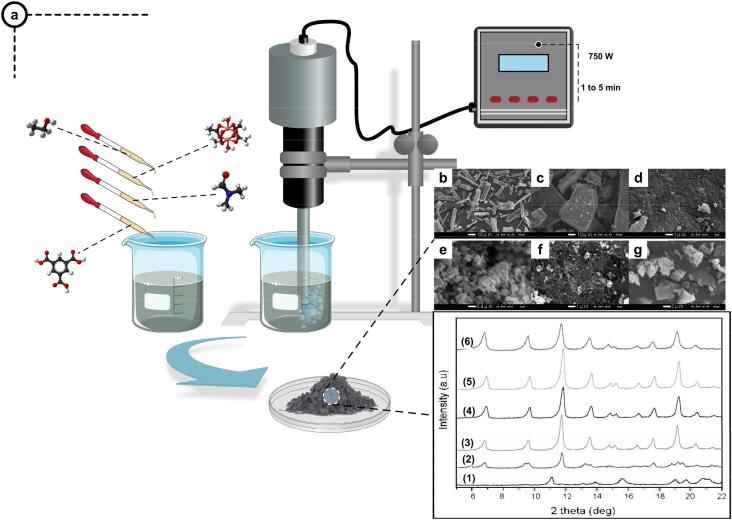
(a) Schematic of Ultrasonic assisted production of Cu-BTC and (b-g) SEM images of MOF synthesized under 1 min sonication with different DMF concentrations. The solvent composition includes water (4 ml), ethanol (1 ml), and DMF((b) to (g) corresponds to 0 to 6 ml DMF addition). Corresponding XRD patterns ((1), (2) refer to six Cu-BTC nanocomposites prepared in different solvent compositions. *(Reproduced with permission from reference*[94]*).*

The application of ultrasonic cavitation for producing MOFs is rather new and was first presented about two decades ago [93]. The effect of ultrasonic irradiation on the Cu-BTC (BTC: benzene-1,3,5-tricarboxylic acid) MOF was first investigated in 2009 by Khan et al. [94]. The proposed technique could significantly reduce the reaction time (as low as 1 min), which was considerably lower than the electric and microwave heating methods. Additionally, the particle size of synthesized MOF remarkably decreased under short-time sonication. On the other hand, increasing the sonication time from 5 to 60 min led to the aggregation of the synthesized MOFs, which is unfavorable. The primary role of solvent (ternary solvent of water + ethanol + DMF) in MOF production yield using the ultrasonic-assisted technique was also reported in this paper. The SEM images of the synthesized MOFs with different solvent concentrations (DMF: 0 to 6 ml) and the corresponding XRD patterns are presented in Fig. 2. It was found that crystal formation is possible even in a small amount of solvent (Fig. 2c), but the BET analysis indicated a decline in the porosity of the obtained materials. SEM images showed that adding 1 to 3 ml DMF is crucial for producing homogeneous fine MOF particles where further increases may cause particle aggregation.

Eight years later, Israr and coworkers synthesized Cu-BTC (BTC = 1,3,5-benzene tricarboxylate) MOF through ultrasonic treatment [95]. Similar to the previous research, the effect of the solvent (binary and ternary mixtures) on the textural properties of Cu-BTC was investigated, and ultrasonic parameters, including sonication time and power, were studied in detail to find the optimum synthetic conditions. Briefly, using a ternary solution of water/ethanol/*N*-dimethylformamide (DMF), sonication time of 120 min, and ultrasonic power of 750 W has led to the highest MOF yield.

**Fig. 3:**
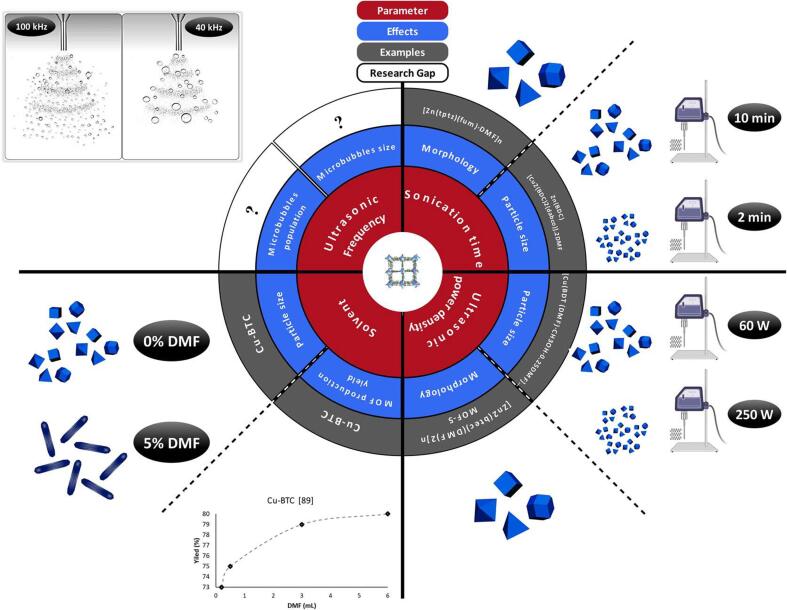
Effects of sonication conditions on the properties of the synthesized MOFs.

The effect of sonication time on the synthesized MOFs has been broadly investigated. Possible effects of ultrasonic parameters on the structural/morphological properties of MOFs is summarized in Fig. 3. Nanocrystals of Cu_3_ (BTC)_2_ were prepared successfully at ambient temperature and under sonication (40 kHz, 60 W, ultrasonic bath) [75]. The experiments were carried out at different sonication times (5, 10, 20, 30, and 60 min), and it was shown that extending the reaction time significantly increased the production yield of MOF (from 62.6 to 85.1 %). Furthermore, the shape and size of the synthesized MOFs were affected by extending the sonication time (9-fold increment in the particle size and shape conversion from spherical to needle-like particles).

However, there was no significant difference in physicochemical properties of the MOF in comparison with those synthesized by the conventional technique. The operational conditions, efficiency analysis, and conclusive results of the most recent researches in which sonication technique was utilized in the MOF production process are summarized in Table 1.

**Table 1:** Operational conditions and major properties of some ultrasonically synthesized MOFs in the literature.

MOF	US freq. (kHz)	US power (W)	US Time (min)	Processing temperature (°C)	Surface area (m^2^/g)	Crystal size (nm)	Pore volume (cm^3^/g)	Yield (%)	Ref.
Cu_3_(BTC)_2_	40	60	5 to 90	Room	1100	10 to 200	0.662	85.1	[75]
Cu-BTC	20	150 to 600	20 to 120	Room	1430	3 to 10 K	0.668	86	[95]
MOF-5	20	20 to 100	10 to 75	129 to 164	3208	5 to 25 K	1.26	nm*	[96]
HKUST-1	nm	nm	30, 60, 120	Post heating @ 120	2025	30–600	nm	79	[62]
TMU-5	nm	350	nm	Post heating @ 80	400.8	195	nm	nm	[97]
Ni-MOFs GO/MOFs	40	200	30	nm	59.8	2 to 50	nm	nm	[98]
Ce-MOF	30	60	30	Room	218.6	60	0.81	nm	[99]
Cu-BTC Cu-BDC ZIF-8 MOF-5	40	100	5 to 60	Room	nm	CU-BDC < 500	nm	nm	[100]
TMU-31 TMU-32	40	360	15 and 30	Room	nm	nm	nm	nm	[101]
Cu-BTC	nm	nm	30	Post-heating @ 80	nm	nm	nm	nm	[102]
Ag- Trimesic acid	24	80	60	Room	170	d^**^ = 35, l^***^ = 85	nm	nm	[103]

* Not mentioned, ** Diameter, *** Length.

Son et al. synthesized MOF-5 in a 50 ml transparent horn-type tube reactor under ultrasonic irradiation [104]. They examined the effect of factors including sonication time (10–75 min), and power density on the textural properties of the synthesized MOF and production yield. The results unveiled the considerable impact of sonication on synthesis time and crystal size. The production time decreased from 24 h to 30 min, and the crystals were 60 times smaller than MOF-5 that was synthesized by the conventional technique.

**Fig. 4:**
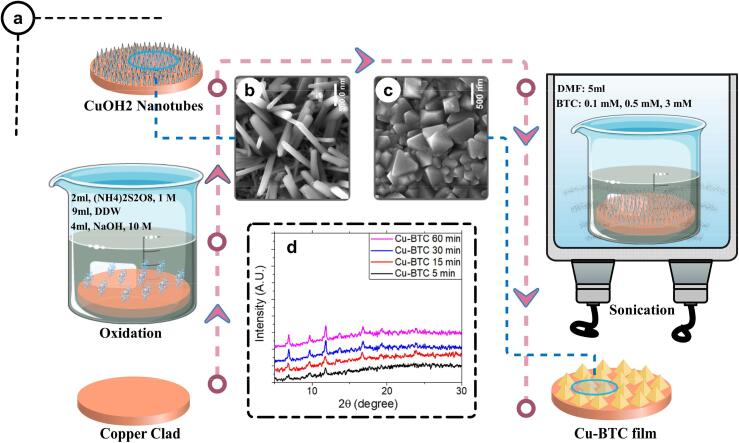
(a) The overall scheme of ultrasonic-assisted in-situ growth of Cu-BTC on the copper clad; (b) SEM images of CuOH_2_ nanotubes on copper clad after oxidation, (c) Cu-BTC MOF after sonication (60 min), and (d) XRD patterns of the produced Cu-BTC MOFs at different sonication times. *(Reproduced with permission from reference*[100])*.*

In addition to the primary method of assisting ultrasonic energy of MOFs production [101], [103], [105], [106], [107], some novel techniques have been reported to reach a more rapid and facile synthesis process. Metal substrates were successfully utilized for the production of Cu-BTC, Cu-BDC, ZIF-8, and MOF-5 films under sonication [100]. A general overview of the applied process for the production of Cu-BTC MOF films is shown in Fig. 4. A two-step sonochemical technique was proposed that includes the formation of nanotubes by oxidizing metal substrates and the synthesis of MOF films in different sonication times (5–60 min). The frequency and power were adjusted at 40 kHz and 100 W, respectively. It was stated that the formation of MOFs could not be progressed without sonication. Furthermore, by increasing the sonication time from 5 to 60 min, the Cu-BDC crystals became well developed and crystalline size increased as well. Additionally, the results of the XRD analyses (Fig. 3d) indicated stronger peaks at longer reaction times, and complete conversion of metal ions to MOF was reached after 30 min sonication. A similar trend was observed in the case of ZIF-8; however, the results for MOF-5 were in contrast with the other three MOFs, where the average crystal size decreased with increasing the sonication time. The complex formation of several nuclei is the main reason behind the observed unusual behavior in MOF-5 synthesis.

In addition to the ultrasonic energy acting as a driving force in the MOF production mechanism, the assisting mechanical waves facilitate the exfoliation of MOF nanosheets [108], [109], [110]. The ultrasonic cavitation breaks the interlayer bonds and increases the exfoliation efficiency based on the selected solvent, ultrasonic frequency, and intensity. Moreover, ultrasonic-driven vibration itself is shown to play a major role in fragmenting MOF nanosheets and separating them from the bulk precursors [104]. Removing the large particles via mechanical methods like centrifugation or sedimentation is the final step of ultrasonic-assisted exfoliation of MOF nanosheets [111]. MOF nanosheets with desired characteristics (thickness, lateral size, and concentration of nanosheets) could be synthesized by precisely controlling the ultrasonic power and energy and selecting a suitable solvent [112].

More recently, some combined methods were introduced to synthesize MOFs by hybrid methods. For instance, Zhao and his colleagues integrated ultrasonication and ball-milling for the high-yield production of nickel-based MOFs [98]. The ball-milling speed rotation was adjusted at 235 rpm, and a sonication frequency of 40 kHz was applied under 200 W power for 30 min. The ultrasonic irradiation facilitated the growth of Ni-MOFs with minimal agglomeration. As a consequence, regular and independent cuboids were formed. On the other hand, ball-milling significantly suppressed grain growth and could effectively refine the crystalline particles.

## 3 MOFs applications for fabrication of mixed matrix membranes

While polymeric membranes are broadly utilized for various separation processes, they suffer from three major drawbacks: (i) fouling/biofouling [113], (ii) low thermomechanical stability, and (iii) the trade-off between permeability and selectivity. Therefore, mixed matrix membranes (MMMs) have emerged to overcome these challenges by incorporating functional nanomaterials into the polymer matrix. The general idea for fabricating MMMs is to induce nanomaterials' thermal, electrical, hydrophilic, antibacterial, and molecular sieve properties to the pristine polymeric membrane. For example, TiO_2_ nanoparticles generate highly oxidizing hydroxyl radicals, which readily attack and decompose organic contaminants in water. Conductive nanomaterials like indium tin oxide (ITO) and antimony tin oxide (ATO) make the membrane electroconductive which can initiate electrochemical reactions upon connection to an external electric field [114]. Rod-shaped or tubal nanomaterials with a high length to diameter ratio may improve the thermomechanical stability of membranes more than spherical nanoparticles [115], [116]. Carbon-based nanomaterials like graphene nanoplatelets, GO nanosheets, carbon nanofibers (CNF), and CNTs can improve hydrophilicity, antibacterial properties, and mechanical strength of membranes [117]. However, the severe aggregation of nanomaterials and their weak compatibility with the polymer matrix have hindered the widespread application of MMMs [13]. Using MOFs offers an exciting opportunity to resolve the compatibility issue as this class of nanomaterials has organic ligands in their structure that could interact better with polymeric materials.

## 4 MOF-based MMMs for water treatment

MOFs have recently attracted significant attention for making water treatment membranes due to their variety and their excellent potential for adjusting the chemical and structural properties of membrane pores. The combination of porosity and functionality of MOFs, and the processability/flexibility of polymeric materials in MMMs, made this hybrid compound an excellent option for water treatment. In a recent study, UiO-66-NH_2_ was incorporated into the matrix of cellulose acetate (CA) polymer, and the resulting MMM was used for the removal of methyl blue (MB) and methyl orange (MO) dyes [118]. Uptake capacity results indicated a significant improvement of synthesized MMMs (480 % for MB, and1150% for MO at 1 ml/min) in comparison to the pristine CA membrane. Moreover, the recyclability tests of the produced MMM showed a constant trend in separation performance (100% removal of MO and MB at 1 ml/ min) after 10 cycles which indicates the high water stability of the MOFs in the membrane matrix. In another study, Meng et al. added BUT-8(A) into the polyethyleneimine (PEI) (25–57 % MOF loading) and coated the mixture on the hydrolyzed polyacrylonitrile (HPAN) substrate [119]. For dye reagents, water permeance between 396 L m^−2^ h^−1^ MPa^−1^ (100 mg L^−1^ methylene blue) to 683 L m^−2^ h^−1^ MPa^−1^ (100 mg L^−1^ congo red) and rejection percentages of 98.3 % and 100 %, respectively (0.5 MPa) were obtained. The improved trend in water permeance and rejections was attributed to the dominance of molecule transportation mechanisms through the channels of MOFs. The fabricated membranes could also reject 9.4 % of MgCl_2_ and 70 % of Na_2_SO_4_ with the corresponding permeance of 100 L m^-2^h^−1^ MPa^−1^ to 300 L m^-2^h^−1^ MPa^−1^. Shu and his coworkers synthesized polymer-compatible nanosheets with a thickness of ca. 3 nm from BUT-203 2D MOF [120]. A thin layer (70nm) of high filler loaded (up to 73 %) MMM was spin-coated on a macroporous hydrolyzed polyacrylonitrile (HPAN) substrate. The filtration performance (permeance up to 870 L m^-2^h^−1^ MPa^−1^ and rejection higher than 97.9 % for anionic dyes) and stability of the MMM over long-term experiments (30 hr) showed the potential of the nano-sized 2D MOFs for separation/purification applications. Besides the positive effects of MOF on permeability/selectivity, they could also be incorporated into the membrane matrix to prevent biofouling because of their superior qualities of containing large surface areas, adjustable pore sizes, and various structural variations. Some MOF-membranes could harm and kill bacteria by the steady and constant release of metal ions [31]. Long-term interactions between bacterial cells and metal ions can be easily facilitated by porous MOFs while the organic ligands of MOFs govern the release of metal ions. Membrane biofouling, or the initial attachment of microorganisms and subsequent growth of cohesive colonies of bacteria on the membrane surface, is considered as the most challenging form of fouling which could seriously diminish the selectivity of these membranes. The permeate quality and membrane permeability may both be severely lowered by extreme biofouling. By utilizing CuBTTri, a water-stable metal–organic framework (MOF), antibiofouling thin-film nanocomposite (TFN) membranes were synthesized by Wen et al. [121]. Observations showed a significant reduction in bacteria (96.7 % reduction for Pseudomonas aeruginosa in compared to the pristine membrane) for the membrane incorporated with only 0.1 % MOF, which showed excellent antibiofouling feature of the synthesized TFN membranes. A lower flux decline of the modified TFN membranes (50 % improvement) over filtration tests confirmed the antibiofouling property of the MOF incorporated membranes.

### 4.1 MOF-based MMMs for gas separation

Gas-phase separation/purification has many applications in both conventional and modern chemical industries, such as alkene/alkane separation, purification of gaseous fuels, and CO_2_ capture (e.g., post-combustion, pre-combustion, oxyfuel process, and direct air capture). The current gas separation membranes are mostly polymeric materials due to their economically and industrially favorable features. Consequently, most efforts in producing industrial-scale gas separation membranes have been devoted to modifying current polymeric materials [122], [123]. However, some major challenges, such as selectivity-permeability trade-off, and plasticization/aging of polymeric membranes under harsh industrial conditions, have urged exploring alternative materials for membrane manufacturing [124], [125]. For their specific and fixed pore sizes and unique topologies, MOFs show great potential for precise molecular-scale sieving and, specifically, gas separation processes [126]. The incorporation of metal–organic frameworks into a polymer matrix to generate a combined form membrane and the deposition of a thin film of the metal–organic framework on a spongy substrate (surface modification) are the two most common methods for introducing metal–organic frameworks into a membrane [127].

There have been reports on major interfacial challenges like poor adhesion, and incompatibility between the polymer matrix and the MOF crystals in the literature [128], [129]. Nevertheless, many researchers have already reported these composite materials with promising separation performance as well as their resistance to plasticization and physical aging, which are very favorable for gas separation applications [57], [130]. While MOF loading could improve the membrane performance, the overloading could be problematic, resulting in mechanical defects and efficiency loss. Also, the weak interfacial interaction between the polymer phase and MOF particles is yet to be resolved [94]. To improve the permselctivity of membranes, MOFs are preferred to be incorporated onto the surface so that the gas stream can be in direct contact with the MOF layer. Rui et al. reported excellent CO_2_ permeance (615 GPU) and selectivity over CO_2_/N_2_ separation (410.7) by seeding IRMOF-1 (Zn_4_O(BDC)_3_) film on an α-alumina substrate [131]. A considerable improvement in CO_2_ permeance (2100 GPU) was observed when a thin layer of a 2D ZnTCPP (zinc(II) tetrakis(4-carboxy-phenyl)porphyrin)) nanosheets was coated on a thin-film composite membrane [132]. Besides the promising permeance results, good mechanical features of the presented composite membrane, facilitate their industrial-scale applications. MOF-membranes have also been utilized to separate a wide range of hydrocarbon-based gas molecules as well as methane, propane, and butane [64], [133], [134].

## 5 Mofs applications for membrane surface modification

Functionalization of membranes with MOFs can be conducted by heterogeneous crystallization on the membrane surface or homogeneous crystallization in the bulk phase, both proceeding under thermal processes. However, other energy sources like microwave and sonication were reported to promote the thermal technique effectively. It was reported that defect-free and uniform MOF-based membranes can be obtained by surface functionalization, while bulk crystallization may lead to defects such as cracks on the membrane surface [135].

**Fig. 5:**
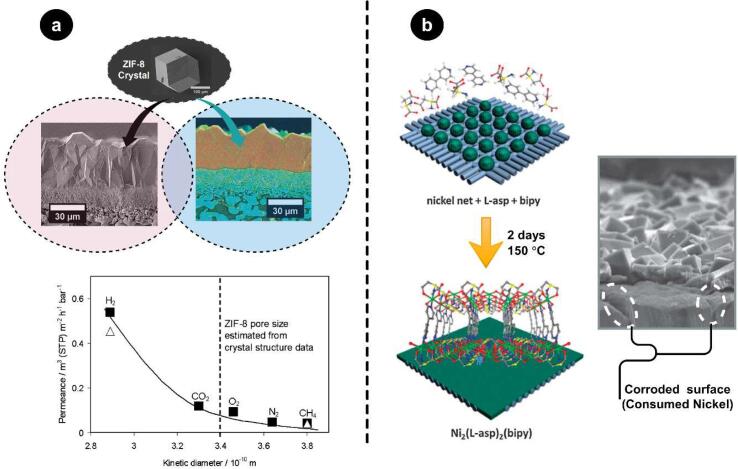
Direct growth of MOFs on membrane surface; (a) SEM (left oval) and EDXS (right oval) images of ZIF-8 on porous titania support, and pore size estimation (bottom curve) using single gas permeance test. (Reproduced with permission from references [137] (b) Scheme of ligand distribution on nickel net, the reacted nickel net with the organic ligands, and the corresponding cross-section SEM image. (Reproduced with permission from references [138]).

For the first time, in-situ growth of polycrystalline MOF-5 as the active layer of a porous alumina membrane was investigated by Liu et al. [136]. Surface area of 2259 m^2^/g and pore size distribution of 1.56 nm were reported for the synthesized MOF nanostructure. Direct growth of MOF crystals on the membrane surface was also carried out by producing ZIF-8 on titania support for gas separation applications. As can be seen in Fig. 5(a), despite the smaller pore size of the synthesized MOF (∼3.4 Å), CH_4_ molecules with a kinetic diameter of 3.8 Å can marginally pass through the composite membrane. Consequently, a smooth cutoff curve, which was formed at 3.4 Å, indicates that ZIF-8, in its nature, is more flexible rather than static [137]. For the first time, in situ growth of homochiral MOF on nickel net was investigated by Kang et al. [138]. A so-called “single metal source” technique was utilized for the formation of Ni_2_(l-asp)_2_(bipy) crystals on a nickel net (Fig. 5(b)). 2-methyl-2,4-pentanediol was used as the chiral molecule to evaluate the performance of the fabricated membrane in the temperature range of 25 to 200 °C under 0.1 MPa. Based on the the enantiomeric excess (ee) values for racemic diol mixtures, the synthesized homochiral MOF membranes were thermaly stable and favorable for chiral resolution application. The ee values increased from 12.9 at 25 °C to 32.5 at 200 °C which is around the boiling point of the selected chiral (2-methyl-2,4-pentanediol). Twofold increase in the permeance of the R enantiomer was detected when the temperature was raised from 25 °C to 200 °C while a slight increase was observed for S enantiomer. This improvement in selectivity was attributed to smaller kinetic diameter of S enantiomers which easily adsorb in the homochiral MOF pores and block the path for diffusion of R enantiomers. By increasing the temperature, as the adsorption of S enantiomer decays, R enantiomers have more chance to diffuse in the resulting free volume, which leads to higher selectivity over R enantiomers than S types.

**Fig. 6:**
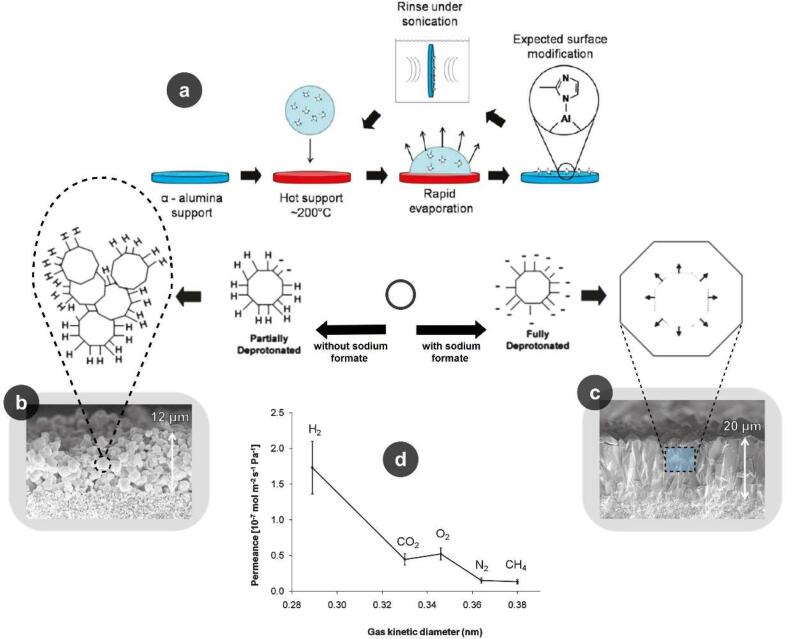
Substrate modification for MOF modified membranes; (a) a schematic overview of the process for covalent linkage of imidazole ligands to the support, (b) cross-section FE-SEM image of ZIF-8 film (grown through the solvothermal technique for 36 h without sodium formate in the solution, (c) cross-section FE-SEM images of ZIF-8 film (grown for 3 h using a methanol solution containing zinc chloride, m-IM, and sodium formate), and (d) single gas permeance test of ZIF-8 (with sodium formate) membranes (Reproduced with permission from reference [139]).

The strength of chemical bonds between MOF particles and membrane surface is the most significant challenge for fabricating robust MOF-functionalized membranes. To deal with this issue in the direct growth method, MOFs, substrates, or both should be modified before the formation of crystals. Surface chemical modification of support material was investigated to improve nucleation in conventional direct growth techniques [139]. Organic ligands of the ZIF-7 and ZIF-8 were used for modifying the α-alumina supports before conducting the solvothermal MOF synthesis process, as schematically depicted in Fig. 6a. ZIF-8 film is synthesized either with or without sodium formate and using different chemicals as the zinc source. Cross-section FESEM (Field Emission Scanning Electron Microscope) images of the films are shown in Fig. 6(b and c). In the presence of sodium formate, the generated layer was found to be continuous with an approximately constant thickness (∼ 20 μm) and larger gently-formed grains (∼ 5–10 μm). However, in the absence of sodium formate, the integrity of generated crystals and homogeneity of the film were both aggravated [139]. The single gas performance of the synthesized membranes is provided in Fig. 6d. The pore window size of 3.4 Å was reported for ZIF-8 nanoparticles, which in the best case led to a selectivity value of 11.6 and 13.0 for H_2_/N_2_ and H_2_/CH_4_, respectively. The fabricated ZIF membranes were reported to be suitable for molecular sieving of small molecules except for CO_2_ and O_2_. The better permeance of O_2_ through this membrane than CO_2_ confirms the greater affinity of O_2_ molecules toward ZIF-8.

**Fig. 7:**
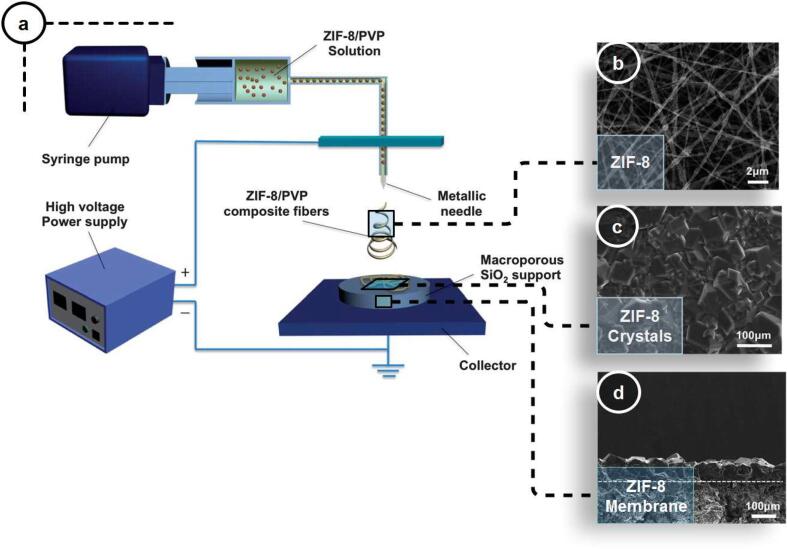
(a) Electrospinning process used as a seeding technique for the synthesis of macroporous ZIF-8 on SiO_2_ wafer. (b) SEM images of electrospun ZIF-8/PVP composite fibers, (c) top surface, and (d) cross-section view of the synthesized ZIF-8 membrane using this technique. (Reproduced with permission from reference [141]).

More recently, seeding techniques for synthesizing MOF membranes have attracted significant attention due to their high controllability of the membrane functionalization process. Electrospinning is one of the best seeding techniques for producing dense and continuous membranes without structural defects, cracks, or intercrystalline gaps [140]. This technique generates nonwoven nanofibers by applying extremely high voltage to an aqueous polymeric solution. Consequently, a microjet stream of the solution is extruded from the nozzle, coating the target substrate. Fan et al. used electrospinning to coat solvothermally synthesized ZIF-8 metal-organic frameworks on SiO_2_ substrate [141]. The scheme of the coating process and SEM images of the membranes are illustrated in Fig. 7. Precise control of the thickness of the seed layer and uniform seed coating on the support resulted in a significant improvement in separation factors compared to the corresponding Knudsen values (H_2_-CO_2_: 55 %, H_2_-CH_4_: 72 %, and H_2_-N_2_: 33 %; 1 atm, 298 K, and 1:1 vol ratio).

## 6 Ultrasonically synthesized MOFs for membrane applications

Ultrasonically synthesized MOF nanocrystals have been used to modify membranes for different applications as summarized in Table. 2. Thompson et al. fabricated ZIF-9/Matrimid (commercially available glassy polyimide) mixed matrix membranes by adding 10 and 25 wt% of the ultrasonically synthesized MOF into the polymer solution and casting the mixture [142].

**Table 2:** Properties of the ultrasonically synthesized MOF-membranes and the corresponding filtration performances.

Support	MOF	Sonication properties: Time/Frequency/power	Filtration process	Permeability-permeation	Rejection/selectivity	Ref.
Matrimid	ZIF-9	Direct sonication: 0–10 min/20 kHz/ 200 W Indirect sonication:0–10 min /40 kHz/ 120 W	CO_2_/CH_4_ Separation	300 barrer	Selectivity: 85 %	[142]
Commercial NF TFC membranes	Cu-MOF	30 min/ 20 kHz/ 100 W	Dye removal	10 LMH	Rejection: Methyl blue:90 % Methyl orange: ∼95 % for	[143]
PVDF	CU-BTC	60 min/ 40 kHz/ 60 W	BSA separation (antifouling test)	185.05 LMH @ 1 bar	Rejection: bovine serum albumin (BSA): 94 %	[144]
PES	Ag-MOF	60 min/ 24 kHz/ 80 W	FO process	@ 2 M NaCl (draw solution): 81 LMH	Js/Jw = 0.13	[145]
PES	Ce-MOF	30 min/ 30 kHz/ 60 W	Dye removal	21.2 LMH	Rejection: Direct Red 16: 99 %	[99]
PS	Cu-BTC	30 min/ nm*/ nm	landfill leachate treatment	190 LMH @ 3 bar	Rejection: landfill leachate (COD: 18000 mg/L): 47 %	[102]
PES	TMU-5	30 min/ nm/ 350 W	powder milk solution (antifouling test)	123.212 @ 3 bar	Rejection: powder milk > 98 %	[97]

* Not mentioned.

**Fig. 8:**
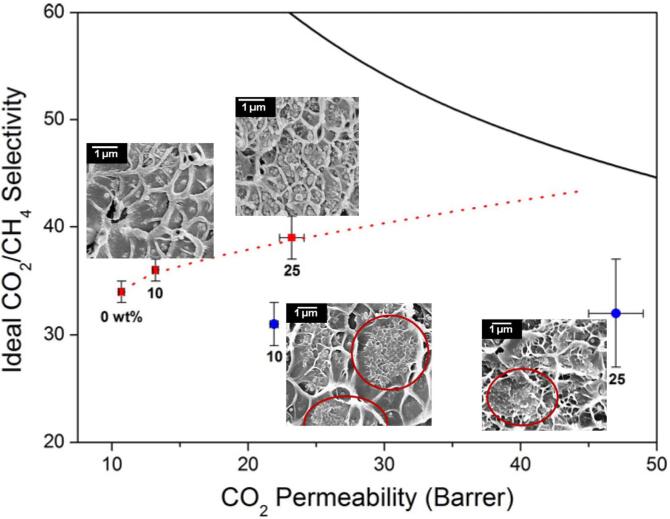
Results of membrane performance tests and the corresponding SEM images: Red: direct sonication; Blue: indirect sonication; Solid line: Upper Bound; Dotted line: Maxwell model predictions. Aggregation sites from indirect sonication are shown with red circles (Reproduced with permission from reference [142]).

Two sonication strategies were investigated for the production of ZIF-9 MOFs. For direct sonication, the solution was sonicated using a sonicator horn with a power output of 200 W and a frequency of 20 kHz. An ultrasonic bath (120 W and 40 kHz) was used for the indirect sonication process. Particle size distribution analysis of the synthesized MOFs showed that increasing the sonication time resulted in the dissolution of the smaller ZIF-8 crystals because of their lower thermodynamic stability and higher surface-to-volume ratio. The disappeared small MOF crystals then rapidly formed big crystals in the nucleation sites to reach the lowest surface energy level (highest stability). Prior to the casting step, ZIF-9/Matrimid solutions were subjected to direct and indirect sonication for dispersing the MOF crystals. Different sonication conditions were executed to evaluate the effect of ultrasound energy intensity on the dispersion of nanocrystalline ZIF-8 MOFs in the polymer matrix. According to the cross-section SEM images (Fig. 8), direct sonication with higher energy intensity (156 W cm^−2^) resulted in a homogeneous distribution of ZIF-8 crystals inside the membrane structure. In contrast, large agglomerations of MOF particles were observed (red circles in Fig. 8)), in the samples synthesized via indirect sonication. Membrane performance tests for fabricated membranes by direct sonication technique resulted in the maximum CO_2_ permeability of 300 barrer (1 barrer = 3.348 × 10–16 mol m/(m^2^.s.Pa)) and selectivity of 85 % (CO_2_/CH_4_) by formation of 25 % ZIF-8 in the membrane matrix.

In a recent study, ultrasonically synthesized Cu-MOF was incorporated into the polydopamine (pDA) layer of pDA-functionalized commercial NF TFC membranes (polyamide selective layer on PES support) [143]. A probe sonicator was utilized at 20 kHz and the power of 100 W to boost the crystal formation reaction. The characterizations revealed that the presence of Cu-MOF nanocrystals in the selective layer could significantly enhance the membrane hydrophilicity, fouling resistivity (∼90 % improvement in flux recovery ratio), and dye rejection (from ∼ 26 % to 90 % for methyl blue, and ∼ 28 % to ∼ 95 % for methyl orange). Sonication was also utilized to produce MOF-PVDF composite membranes using MOF-199 (CU-BTC) for microfiltration tests (TMP: 0.1 MPa) [144]. The incorporation of MOF increased the water flux from 12.75 LMH for the pristine membrane to 185.05 LMH and the bovine serum albumin (BSA) rejection was maintained at a high level ( 94 %). Also, the MOF-PVDF membranes showed consistent pure water flux of 95 Lm^-2^h^−1^ after 40 days, indicating their antifouling properties. The antibacterial property of the pristine and modified membranes was also investigated by using *Staphylococcus aureus* (*S. aureus*) and E. coli bacteria. The results showed a clear reduction in the number of colonies by increasing the wight fraction of the incorporated MOF-199. For *S. aureus* the number of colonies was decreased from 4 to 0, by increasing the MOF from 0 to 5 wt%, respectively.

**Fig. 9:**
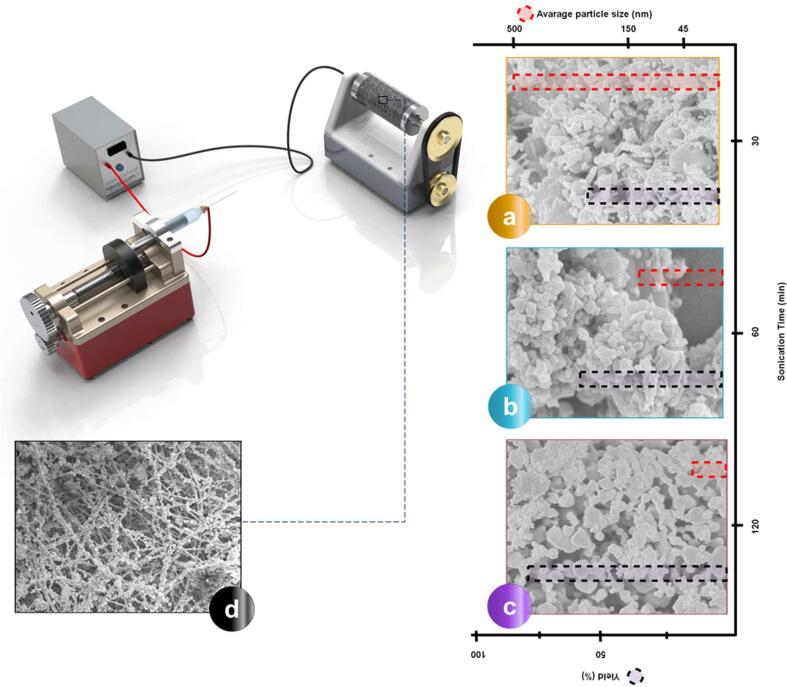
Schematic electrospinning of HKUST-1 hybridized PAN nanofibers using ultrasonically synthesized HKUST-1 particles at different sonication times. SEM images of MOF crystals; (a) 30 min, (b) 60 min, (c) 120 min, and (d) MOF-fiber conjugation after three-hour continuous electrospraying. *(Reproduced with permission from reference*[62]*).*

Sonication (30–120 min) was also used for the production of HKUST-1 crystals (30–200 nm) [62]. A solution of HKUST-1 embedded polyacrylonitrile (PAN) in DMF solvent was then electrospun to form nanofibrous membranes. BET results of the membranes revealed a surface area of 1217 m^2^ g^−1^, and a pore volume of 0.53 to 0.56 cm^3^ g^−1^. The synthesized membranes were then used for carbon dioxide separation experiments. A significant reduction in MOF size from 500 nm to 45 nm was reported by increasing the sonication time from 30 to 120 min. However, BET results indicated a clear reduction in the surface area and the volumetric capacity of the synthesized MOFs when sonication time exceeds 60 min. This observation was justified by the results from the XRD test. Some clear and well-defined peaks were specifically formed for 1hr sonicated MOFs samples, which showed the presence of more crystalline mixed phases when compared to the other samples. Further details about the effect of sonication time on MOF size and MOF adsorption yield (N_2_) are summarized in Fig. 9. The experimental data also showed the superiority of the sonochemical technique for the production of HKUST-1 over the conventional solvothermal method in terms of particle size (0.1–0.2 µm vs 2–6 µm), and surface area (2025 m^2^ g^−1^ vs 1095 m^2^ g^−1^). Additionally, XRD patterns of the ultrasonically synthesized MOFs indicated sharper peaks and, thus, more crystalline mixed phases. Substantial surface area and excellent pore volume of the electrospun HKUST-1 (up to 651 cm^3^ g^−1^ adsorption for N_2_) significantly improved the adsorption capacity of the membranes decorated with ultrasonically synthesized MOFs to 412.23 cm^3^ g^−1^ compared with 180 cm^3^ g^−1^ for the solvothermally synthesized MOF-loaded fiber membrane with same testing conditions. Finally, thermogravimetric analysis (TGA) of the MOF-based membranes confirmed their good thermal stability (decomposition temperature of 270 °C), allowing their application in harsh thermal conditions.

Ultrasonic-assisted synthesized MOFs were also used to promote the key features of thin-film composite (TFC) membranes in the forward osmosis (FO) process [145]. Nano-seized MOF crystals with Ag ion clusters and benzene tricarboxylic acid ligands were synthesized using 24 kHz ultrasonic waves, an energy level of 80 W, and a pulse mode of 0.6 for 60 min. DLS analysis indicated the Gaussian hydrodynamic diameter distribution of around 33 nm for the synthesized MOF nanoparticles. Furthermore, when sonication was carried out in hexane, an obvious decrease in the length of the MOF nanorods was observed. Shorter MOF nanorods resulted in a homogeneous and defect-free surface which facilitated MOF incorporation onto the active layer of synthesized TFC membranes. The observations indicated that an appropriate loading of the US-assisted synthesized MOF provided a lower transport resistance in thin-film membranes' active layer while preserving its permeation selectivity. XPS results showed a lower crosslinking density of the selective layer after incorporating the MOF, likely leading to less transport resistance. Besides, the incorporation of MOF in the active layer reinforced the attraction of water molecules through hydrogen bonding originated from hydrophilic functional groups and promoted the water permeability through the barrier layer. The solute/water permeability ratio decreased by adding MOF up to 0.04 w%/v; however, further loading increased the ratio, implying that 0.04 w%/v is the optimum loading for fabricating MOF-based FO membranes.

Mohammadnezhad et al. investigated the effects of using cerium-based MOFs ([Ce(tp)(NMP)_2_(CH_3_COO)]_n_) in the matrix of polyethersulfone (PES) for nanofiltration application (milk powder solution) [99]. Nanocrystalline MOFs were firstly synthesized under ultrasonic power of 60 W for 30 min at ambient temperature. The Ce-MOF/PES mixed matrix membranes were synthesized using the immersion precipitation phase inversion technique with 0 to 1 % MOF, 18 % PES, and 1 % PVP in DMAc solvent. The size distribution histogram of the synthesized Ce-MOFs indicated the average nanocrystalline size of 80 nm. The water contact angle analysis showed a 15 % decrease in water contact angle (from 63.2° to 53.5°) by adding 1 wt% of Ce-MOF to the membrane matrix. Pure water flux of the synthesized MMMs increased by 44 % by adding MOF up to 0.1 wt% (from 14.69 to 21.2 Kg m^-2^h^−1^), owing to the increased wettability of membranes. Further addition of MOF up to 1 wt% decreased water flux likely due to the pore blocking and reduced porosity. Moreover, the nanofiltration tests on Direct Red 16 revealed up to 99 % rejection compared to 86 % for the pristine PES membrane by just adding 0.1 wt% Ce-MOF.

More recently, ultrasonically synthesized copper-based MOFs (Cu-BTC) were incorporated into polysulfone membranes and used for the landfill leachate treatment process [102]. 1, 3, 5-benzenetricarboxylic acid (H_3_BTC), and copper (II) nitrate trihydrate were dissolved in dimethylformamide (DMF) solvent and the mixture was then sonicated for 30 min under atmospheric conditions before the next steps. The synthesized MOFs were dispersed in an organic solvent (NMP) and sonicated for 1 h. After adding the main polymer, surfactant, and pore former, the solution was re-sonicated for another 1 h. Afterward, the bubble-free solution was cast onto polyester non-woven fabric and then submerged into a coagulation bath. By incorporating 2 wt% of ultrasonically synthesized Cu-BTC MOFs into polysulfone membranes, the pure water flux and the landfill leachate flux increased by 70 % and 50 %, respectively. The presence of carboxylic groups in the synthesized MOFs improved the membrane wettability as the water contact angle decreased from 65.4 °to 56.7° The 24hr filtration results on sodium alginate indicated a significant improvement in the antifouling performance of the MMMs. 40 % less decline was observed in comparison to the neat membrane. The sonication method was also used to produce TMU-5 metal–organic frameworks at 350 W in continuous mode. The synthesized MOFs were then blended at 0.1, 0.5, and 1 wt% with polyethersulfone (PES) to form PES/TMU-5 mixed matrix membranes [97]. 800 mg.L^-1^ powder milk solution was used as a feed to evaluate the antifouling performance of the synthesized membrane in the pressure range of the ultrafiltration process (3 bar). According to the water contact angle results, the hydrophilicity of the MOF-PES membrane increased up to 23.2 % by adding 1 wt% of TMU-5 to the PES matrix. The optimum pure water and powder milk water fluxes and antifouling property of the MOF-based membranes were achieved at 0.1 wt% loading. Exceeding this concentration has severely decreased the fluxes and slightly affected the fouling resistance of the membranes. The flux reduction was attributed to pore blocking of the dense active layer by the surplus of TMU-5 nanoparticles.

## 7 Conclusions and future outlook

Crystalline porous MOF are a very interesting family of materials to be used in various separation processes. The MOF materials benefit from excellent features like uniform pore distribution which can be tuned in chemical composition and geometry. Based on the results in the literature, the grain size of ultrasonically synthesized MOFs is mostly smaller than those produced by conventional thermal techniques; consequently, these MOFs act more efficiently as a modifier agent and has smaller defects in membrane production processes. Adding MOF crystals into the membrane matrix led to a considerable improvement in membrane hydrophilicity/wettability (more hydrogen bonding) which results in substantial improvements in pure water flux. Furthermore, observations indicated that the membrane surface area/roughness can be elevated by surface modification of proper MOFs. There are only a few papers that studied the effect of ultrasonic parameters (i.e. sonication time, power, frequency, and sonication mode) on the performance of MOF-modified membranes in the liquid and gas phase separation/purification processes. The key results of this review can be summarized as follows:

The crystallization time of MOFs can be reduced significantly when the ultrasonication technique was employed.

The effects of frequency, power, and sonication mode (continuous or pulsed) have not been sufficiently investigated.

Compared to the conventional thermal techniques, MOFs with smaller crystal sizes can be obtained when the ultrasonic-assisted technique is utilized.

The produced MOFs from the sono-synthesis method increases their crystallinity (excellent physical characteristics like polyhedral grain shapes with sharp edges and smooth faces) and correspondingly higher porosity and surface area could be expected.

Membrane hydrophilicity and correspondingly water flux were mostly increased when they were modified through smaller and finer ultrasonically synthesized MOFs.

Drastically elevated temperature/pressure in microscale zones powered by sonication can be deemed a convenient technique for the non-thermal synthesis of nanomaterials. Although sonication leads to slight increase in the temperature of the solution, it would not be considered as thermal technique. Ultrasonic-assisted synthesis of MOFs is not a new topic and the effect of the sonication on the characteristics of the final MOF crystals has been investigated in different research works. However, in the majority of the presented works, among the sonication parameters such as frequency and intensity, only the effect of sonication time was investigated. It was revealed that physical characteristics of the synthesized MOFs, e.g., size and shape, which are governed by their production technique, play a key role in their effectiveness. Despite the undeniable influence of sonication on crystalline properties of MOFs, there is just a limited number of researches in which sonication parameters are investigated in detail. Therefore, an obvious research gap exists in a perfect understanding of the sonication mechanism and all-controlling parameters in the final MOF membranes, and further studies and experiments are still required to be done in this regard.

## CRediT authorship contribution statement

**Amirhossein Taghipour:** Conceptualization, Investigation, Writing – original draft, Writing – review & editing. **Ahmad Rahimpour:** Investigation, Project administration, Supervision. **Masoud Rastgar:** Conceptualization, Investigation, Supervision, Writing – review & editing. **Mohtada Sadrzadeh:** Funding acquisition, Investigation, Supervision, Writing – review & editing.

## Declaration of Competing Interest

The authors declare that they have no known competing financial interests or personal relationships that could have appeared to influence the work reported in this paper.

## Data Availability

No data was used for the research described in the article.
